# Prevalence and risk factors of helicobacter pylori in Turkey: a nationally-representative, cross-sectional, screening with the ^13^C-Urea breath test

**DOI:** 10.1186/1471-2458-13-1215

**Published:** 2013-12-21

**Authors:** Nilufer Ozaydin, Sinan A Turkyilmaz, Sanda Cali

**Affiliations:** 1 Department of Public Health, School of Medicine, Marmara University, Istanbul, Turkey; 2 Institution of Population Studies, Hacettepe University, Ankara, Turkey

**Keywords:** Helicobacter pylori prevalence, Risk factors of helicobacter pylori infection, Smoking, Alcohol use

## Abstract

**Background:**

Helicobacter pylori is an important global pathogen infecting approximately 50% of the world’s population. This study was undertaken in order to estimate the prevalence rate of Helicobacter pylori infections among adults living in Turkey and to investigate the associated risk factors.

**Method:**

This study was a nationally representative cross sectional survey, using weighted multistage stratified cluster sampling. All individuals aged ≥18 years in the selected households were invited to participate in the survey. Ninety two percent (n = 2382) of the households in 55 cities participated; 4622 individuals from these households were tested with the ^13^C-Urea breath test. Helicobacter pylori prevalence and associated factors were analysed by the t test, chi square and multiple logistic regression with SPSS11.0.

**Results:**

The weighted overall prevalence was 82.5% (95% CI: 81.0-84.2) and was higher in men. It was lowest in the South which has the major fruit growing areas of the country. The factors included in the final model were sex, age, education, marital status, type of insurance (social security), residential region, alcohol use, smoking, drinking water source. While education was the only significant factor for women, residential region, housing tenure, smoking and alcohol use were significant for men in models by sex.

**Conclusion:**

In Turkey, Helicobacter pylori prevalence was found to be very high. Individuals who were women, elderly adults, single, had a high educational level, were living in the fruit growing region, had social security from Emekli Sandigi, were drinking bottled water, non smokers and regular alcohol consumers, were under less risk of Helicobacter pylori infection than others.

## Background

Helicobacter pylori was first discovered in 1983, and eleven years later in 1994 the International Agency for Research on Cancers (IARC) classified H.pylori as a definite class 1 carcinogen [1,2]. It is a small, spiral, gram-negative bacillus which inhabits the mucus layer overlying the gastric epithelial cells in humans. It produces a potent urease. The isolation of H.pylori from the human gastric mucosa and the demonstration of its involvement in gastritis, peptic ulcer disease and gastric cancers have radically changed our perception of these diseases. Development of atrophy and metaplasia of the gastric mucosa are strongly associated with H.pylori infection [2-5].

The greatest risk for infection appears to be during childhood and early adult years [6]. Although infected individuals often have histological evidence of gastritis, the vast majority of infections are asymptomatic [2]. Current evidence indicates that disparate disease outcomes are not related solely to the genetic diversity of H.pylori, but also to host factors and environmental agents [7]. Further delineation of the host response to infection, to specific environmental exposures or to bacterial virulence factors is required to identify which patients infected with H.pylori are at greatest risk of developing disease. Identifying and understanding such interactions should promote the development of optimal outcomes.

H.pylori is a public health problem in both developed and developing countries [8]. The IARC has stressed that the need for effective, population based screening programs is essential for tackling cancer [9].

Most previous studies have been carried out in clinical settings on small samples. There is limited evidence concerning the prevalence, determinants and mode of infection in representative population samples. This is the first population based study of a country-wide representative sample with a high response rate using the most sensitive and specific test the ^13^Carbon Urea Breath Test (^13^C-UBT) to have been carried out in Turkey. The aim of this survey was to estimate the prevalence rate of H.pylori infection among adults aged ≥18 years and to investigate the factors associated with an H.pylori infection in Turkey.

## Methods

### Study population

A study of the prevalence and risk factors *of H.pylori* infection in Turkey (TURHEP) was a nationally representative, population based cross-sectional screening with the ^13^C-Urea Breath Test. A weighted, multistage, stratified cluster sampling approach was used in the selection of the sample. For this study, 100 different residential areas were selected as clusters for an optimal distribution with a target sample size of 2500 selected households based on the results of the General Population Count of Turkey held in 2000 (Additional file 1: Figure S1). Households which were to be visited in each cluster were selected randomly by the Turkish Statistical Institute.

**Figure 1:**
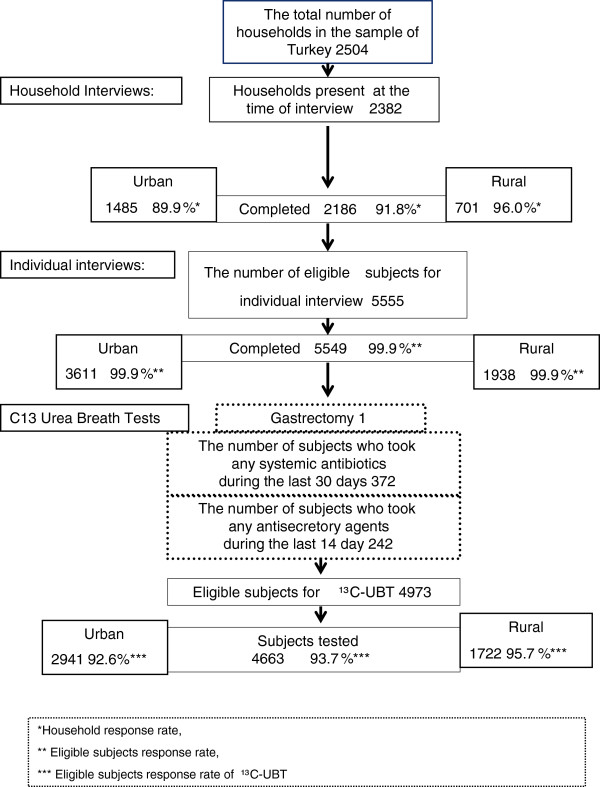
Flowchart of TURHEP study in Turkey.

The eligible individuals were all those aged ≥ 18 who had been present in the selected household on the night before the day of the visit. Among the individuals interviewed those who had undergone a gastrectomy, who had used antibiotics during the preceding 30 days or who had used any proton pump inhibitors during the preceding 14 days were excluded from the survey. The next step was the performance of the ^13^C-UBT on those who accepted this test [10,11] (Figure 1).

### Breath sample collection

At the first visit, eligible and willing people were informed about a required minimal six-hour period of fasting. At the second visit, after ensuring that they had fasted, two breath samples were collected as first samples. The test solution, 75 mg ^13^C-urea in 30 ml drinkable water (Helicobacter Test INFAI, Germany), was given after 200 ml of standard orange juice had been drunk. Thirty minutes later two breath samples were taken. Samples were measured by isotope ratio mass spectrometry (IRMS) in Istanbul between August 2003 and February 2004.

The test results were evaluated as H.pylori-negative when the ^13^C difference between 0th minute sample and 30th minute sample was lower than 4.00 and as H.pylori-positive when it was equal to or higher than 4.00.

### Ethical issues

The study protocol was reviewed and approved by the Research Ethics Committee of the School of Medicine of Marmara University. All participants signed a written informed consent.

IRMS measurements were performed in the University and a trained technician employed by Marmara Health Education and Research Foundation measured the samples during the period of the study.

### Variable definitions

The primary outcome variable, the results of the H.Pylori UBT were categorized as positive or negative. Demographic variables (age, sex, residential region, geographic region, marital status, education), economic status (occupation, social security status ^1a^(Emekli Sandigi, SSK, BAG-KUR, Green Card, private insurance, foreign insurance or none), housing tenure, environmental condition (number in household, bedrooms, source of drinking water, type of toilet system, source of heating) in or out of the home as well as cigarette and alcohol consumption were considered in the analysis.

The geographic regions defined five major regions of the country (West, South, Central, North and East) (Additional file 1: Figure S1).

### Statistical methods

All analyses incorporated sampling weights that were adjusted for the complex study design of TURHEP.

The characteristics of H.pylori-positive and H.pylori-negative participants were compared using the chi-square test for categorical variables and the two sample t-test for continuous variables.

Odds ratios (OR) and 95% confidence intervals (CI) for the association between *H.Pylori* infection and each potential risk factor were estimated using multivariable logistic regression models. The covariates included in the models were those significantly associated with *H.Pylori* in the univariate analyses (p < 0.05). The group presenting the lowest infection risk was chosen as the indicator. The final model was developed using a stepwise procedure with backward elimination, with inclusion and exclusion criteria set at the significance level of 0.05 and 0.10 respectively. The multiple logistic regression model fit was determined by the Hosmer-Lemeshow test statistic. A model fits the data if the Hosmer-Lemeshow statistic has a p > 0.05. Significant predictors were identified and ORs calculated with 95% CIs. The following variables were considered in the model: sex, age, region (West, South, Central, North, East), residence (urban, rural), marital status (never married, currently married, widowed/divorced), education level (no education, primary complete, secondary complete, high school +), type of insurance (Emekli Sandigi, private/foreign, BAG KUR, SSK, none, green-card), occupation (employed/unemployed) housing-tenure (owned by a household-member, lodging/no-rent paid), household population per bedroom, source of drinking-water (bottled-water, piped-water, public-fountain, others:‘river/rain-water/etc.’, smoking (never, current-nonsmoker, current-occasional-smoker, regular-smoker) and alcohol consumption (regular drinker, current occasional-drinker, current non-drinker, never).

## Results

In TURHEP, 2382 households in 100 clusters from 55 cities (Among 81 cities) were available for interview (Additional file 1: Figure S1) and 91.8% were successfully interviewed. The household response rate for urban areas were 89.9% and for rural areas 96.0% (Table 1, Figure 1). The main reasons that the field teams were unable to interview was that some of the houses were vacant at the time of the interview or household members were away for an extended period.

**Tables 1:** Results of the household, individual interviews and breathe samples

	**Residence**		
**Results**	**Urban**	**Rural**	**Total**
**Household Interviews**			
Dwellings sampled	1753	751	2504
Households found	1652	730	2382
Households interviewed	1485	701	2186
**Household Response Rate (%)**	**89.9**	**96.0**	**91.8**
**Individual Interviews**			
Eligible individual	3616	1939	5555
Eligible individual interviewed	3611	1938	5549
**Eligible Individual Response Rate (%)**	**99.9**	**99.9**	**99.9**
^**13**^**C-UBT’s**			
Number of people who had gastrectomy	0	1	1
Number of the people who had antibiotic treatment during the last 30 days	294	78	372
Number of people who used PPI during the last 14 days	174	68	242
Eligible people for test	3174	1799	4973
Eligible people tested	2941	1722	4663
**Eligible people tested rate (%)**	**92.6**	**95.7**	**93.7**

Among 5555 eligible individuals in households, 5549 were successfully interviewed (99.9%). The total number of eligible people for breath test was 4973 and of these 4663 breath samples were collected (93.7%). Three hundred and seventy two individuals who had used antibiotic therapy for any reason during the last 30 days, 242 individuals who had used proton- pump inhibitors during the last 14 days and 1 person who had had a gastrectomy were excluded. The main reason for failure to collect breath samples from the eligible people was that they could not stand the 6-hour fast or were unwilling to undertake the 6-hour fast. Also, a number of eligible individuals were obliged to be outside or working after 6 hrs and a few people did not agree to give breath samples although they gave no reason.

Of the 4663 breath samples, 4622 were measured (99.1%). Forty one breath-samples could not be measured for technical reasons (Table 1).

### The basic socio-demographic characteristics and H. pylori infection

The H.pylori infection prevalence was 82.5% in the population aged ≥18. It was more prevalent in men than women after controlling for confounding factors (Tables 2 and 3, Additional file 2: Figure S2). There was an inverse association between age and *H.pylori* infection (OR:0.98, 95%CI 0.97-0.99) (Tables 2 and 3). Those living in Central or Eastern Turkey were more at risk than those living in Southern Turkey (Tables 2 and 3, Additional file 2: Figure S2).

**Table 2:** Socio-demographic factors associated with Helicobacter pylori infection

**Socio-demographic factors**	**Hp positive**		**Hp negative**			
	**n**	(%)*	**n**	(%)*	**Total**	P**
**Sex**						
Female	2075	(81.4)	457	(18.6)	2532	0.014
Male	1777	(83.9)	313	(16.1)	2090	
**Age groups**						
18–24	736	(79.6)	170	(20.4)	906	0.000
25–34	957	(86.3)	145	(13.7)	1102	
35–44	746	(84.2)	123	(15.8)	869	
45–54	599	(83.7)	108	(16.3)	707	
55–64	372	(78.9)	99	(21.1)	471	
65 +	442	(78.6)	125	(21.4)	567	
**Region**						
West	1027	(80.3)	247	(19.7)	1274	0.000
South	444	(78.7)	118	(21.3)	562	
Central	1089	(85.0)	192	(15.0)	1281	
North	369	(82.3)	83	(17.8)	452	
East	923	(88.1)	130	(11.9)	1053	
**Residence**						
Urban	2411	(81.7)	509	(18.3)	2920	0.020
Rural	1441	(84.0)	261	(16.0)	1702	
**Total**	**3852**	**(82.5)**	**770**	**(17.5)**	**4622**	

*weighted , **p based on X^2^ test.

**Table 3:** Adjusted odds ratios for Helicobacter pylori positivity for various risk factors in final model

**Variable and categories**	**B**	**P**	**OR**	**95% CI**	
**Sex**					
Female			1.0		
Male	0.217	0.035	1.242	1.015,	1.519
**Age**	-0.015	0.000	0.986	0.979,	0.992
**Education**					
High school +			1.0		
No education	0.484	0.003	1.623	1.176,	2.239
Primary complete	0.511	0.000	1.666	1.333,	2.083
Secondary complete	0.405	0.013	1.499	1.091,	2.059
**Marital status**					
Never married			1.0		
Widowed/Divorced	0.425	0.030	1.530	1.042,	2.246
Currently married	0.554	0.000	1.739	1.378,	2.197
**Social security**					
Emekli sandigi			1.0		
Private/foreign	-0.532	0.187	0.587	0.266,	1.295
BAG-KUR	0.009	0.952	1.009	0.757,	1.345
SSK	0.450	0.001	1.568	1.213,	2.026
None	0.439	0.002	1.550	1.174,	2.048
Green Card	0.383	0.077	1.467	0.960,	2.241
**Source of drinking water**					
Bottled water/demijohn/pet water			1.0		
Piped water (in house/garden/outside)	0.572	0.000	1.772	1.404,	2.236
Spring/public fountain	0.517	0.002	1.677	1.218,	2.308
Other (river. rain water etc.)	0.495	0.008	1.640	1.135,	2.371
**Smoking**					
Never			1.0		
Tried at past. currently non-smoker	0.038	0.750	1.039	0.821,	1.316
Tried at past. currently occasional smoker	-0.003	0.985	0.997	0.703,	1.413
Regular smoker	0.350	0.005	1.419	1.113,	1.808
**Alcohol**					
Regular consumer			1.0		
Tried at past. currently drinking occasionally	0.586	0.028	1.798	1.066,	3.032
Tried at past. currently non-drinker	0.687	0.012	1.988	1.161,	3.403
Never	0.692	0.010	1.997	1.182,	3.374
**Region**					
South			1.0		
West	0.186	0.147	1.204	0.937,	1.549
North	0.172	0.343	1.188	0.832,	1.696
Central	0.382	0.007	1.466	1.111,	1.934
East	0.563	0.001	1.756	1.264,	2.439
**Constant**	-0.473	0.173	0,623		

Variables entered in the model: sex, age, residence, region, marital status, education, social security, occupation, housing tenure, source of drinking water, the number of the household per sleeping room, smoking, and alcohol consumption.

### Socio-economic status and H.pylori infection

A current *H.pylori* infection was associated with education, social-security status and water supply (Tables 4 and 3). Occupation, the number in the household, the source of heating and the total monthly family income were not in the final logistic regression models. Housing tenure was the only significant factor in the men’s model (Table 5). There was an inverse association of educational level and H.pylori infection; individuals with lower educational levels had a higher risk than high school graduates and those with a higher education.

**Table 4:** Socio-economic factors associated with Helicobacter pylori infection

**Socio-economic Factors**	**Hp positive**		**Hp negative**			
	**n**	(%)*	**n**	(%)*	**Total**	**p**
**Education** (n = 4577)						
No education	747	(82.6)	163	(17.4)	910	0.000
Primary complete	1801	(86.0)	276	(14.0)	2077	
Secondary complete	355	(85.2)	59	(14.8)	414	
High school +	913	(75.4)	263	(24.6)	1176	
Social security** (n = 4573)						
SSK	1266	(83.9)	228	(16.1)	1494	0.000
Emekli sandigi	431	(74.5)	139	(25.5)	570	
BAG-KUR	546	(78.5)	148	(21.5)	694	
Green Card	324	(87.2)	42	(12.8)	366	
Private/foreign	21	(63.9)	10	(36.1)	31	
None	1223	(85.4)	195	(14.6)	1418	
**Housing tenure** (n = 4597)						
Owned by a household member	2761	(81.6)	583	(18.4)	3344	0.05
Rented	762	(84.5)	134	(15.5)	896	
Lodging/no money paid	306	(85.3)	51	(14.7)	357	
**Occupation** (n = 4503)						
Agriculture & animal husbandry	516	(86.0)	83	(14.0)	599	0.000
Industry	228	(86.0)	35	(14.0)	263	
Construction	111	(92.7)	9	(7.3)	120	
Service	360	(78.7)	87	(21.3)	447	
Housewife/retired/unemployed	522	(78.7)	120	(21.3)	642	
Other	2021	(82.9)	411	(17.1)	2432	
**Household population**						
1–3 person/home	1000	(79.3)	250	(20.7)	1250	0.000
4–5 person/home	1592	(81.4)	334	(18.6)	1926	
6 + person/home	1260	(87.3)	186	(12.7)	1446	
**Rooms for sleeping** (n = 4575)						
1–2	2354	(82.2)	482	(17.8)	2836	0.06
3–4	1382	(82.5)	277	(17.5)	1659	
5 +	73	(93.0)	7	(7.0)	80	
**Source of the drinking water** (n = 4594)						
Piped water (in house/garden/outside)	2342	(83.6)	445	(16.4)	2787	0.00
Spring/public fountain	726	(83.8)	141	(16.2)	867	
Bottled water/demijohn/pet water	327	(72.9)	109	(27.1)	436	
Other ((river, rain water etc.))	430	(85.4)	74	(14.6)	504	
**Type of toilet system** (n = 4598)						
Connected to drainage system	2778	(82.6)	551	(17.4)	3329	0.76
Closed pit	1033	(81.9)	215	(18.1)	1248	
Other (No facility)	18	(86.7)	3	(13.3)	21	
**Source of heating** (n = 4553)						
Radiator (Central heating)	366	(75.0)	102	(25.0)	468	0.00
Radiator (Private)	147	(71.0)	62	(29.0)	209	
Natural gas stove	83	(84.8)	14	(15.2)	97	
Stove (Cool/wood)	2985	(84.4)	536	(15.6)	3521	
Animal excrement	103	(86.4)	17	(13.6)	120	
Electricity	85	(83.7)	18	(16.3)	103	
Gas stove	28	(78.0)	7	(22.0)	35	
**Family income** (USD/month)*** (n = 4194)						
14–179	874	(84.6)	147	(15.4)	1021	0.00
183–394	1472	(83.7)	272	(16.3)	1744	
398–538	503	(83.8)	103	(16.2)	606	
541 +	632	(75.1)	191	(24.9)	823	
Total	3852	(82.5)	770	(17.5)	4622	

*Weighted, **The group (disabled / orphan hood payment by government, n = 29) is excluded. ***1 USD = 1 395 000 TL.

**Table 5:** Adjusted odds ratios for Helicobacter pylori positivity for various risk factors by sex

	**Sex**									
	**Men**					**Women**				
**Variables**	**B**	**P**	**OR**	**CI 95%**		**B**	**P**	**OR**	**CI 95%**	
**Age**	-0.013	0.009	0.987	0.977,	0.997	-0.013	0.005	0.987	0.978,	0.996
**Education**										
High school +								1.0		
No education						0.813	0.000	2.254	1.517,	3.350
Primary complete						0.762	0.000	2.142	1.593,	2.880
Secondary complete						0.679	0.006	1.971	1.213,	3.203
**Marital Status**										
Never married			1.0					1.0		
Widowed/Divorced	0.450	0.242	1.568	0.738,	3.335	0.294	0.209	1.342	0.848,	2.122
Currently married	0.551	0.005	1.735	1.182,	2.547	0.537	0.000	1.712	1.272,	2.304
**Social Security**										
Emekli sandigi			1.0					1.0		
Private/foreign	0.541	0.428	1.718	0.451,	6.546	-1.216	0.023	0.296	0.104,	0.848
BAG-KUR	0.385	0.083	1.469	0.951,	2.269	-0.186	0.339	0.831	0.568,	1.215
SSK	0.768	0.000	2.155	1.478,	3.140	0.276	0.110	1.317	0.940,	1.847
None	0.894	0.000	2.444	1.632,	3.661	0.178	0.340	1.194	0.829,	1.720
Green Card	0.593	0.068	1.809	0.956,	3.422	0.302	0.290	1.353	0.773,	2.369
**Housing Tenure**										
Owned by household members			1.0							
Rented	0.276	0.109	1.317	0.940,	1.845					
Lodging/no money paid	0.692	0.034	1.997	1.055,	3.779					
**Source of Drinking Water**										
Bottled water/demijohn/pet			1.0					1.0		
Piped water	0.418	0.023	1.518	1.060,	2.174	0.684	0.000	1.981	1.477,	2.657
Spring/public fountain	0.073	0.763	1.076	0.669,	1.728	0.901	0.000	2.461	1.644,	3.684
Other (river. rain water etc.)	0.808	0.012	2.244	1.198,	4.204	0.438	0.050	1.550	1.001,	2.402
**Smoking**										
Never			1.0							
Tried at past. currently non-smoker	0.167	0.381	1.182	0.813,	1.719					
Tried at past. currently occasional smoker	-0.073	0.792	0.929	0.539,	1.602					
Regular smoker	0.449	0.017	1.566	1.083,	2.264					
**Alcohol**										
Regular consumer			1.0							
Tried at past. currently drinking occasionally	0.731	0.009	2.078	1.202,	3.590					
Tried at past. currently non-drinker	0.764	0.009	2.148	1.214,	3.799					
Never	0.779	0.007	2.180	1.243,	3.822					
**Region**										
South			1.0							
West	0.423	0.032	1.526	1.037,	2.246					
North	0.451	0.118	1.570	0.891,	2.764					
Central	0.653	0.002	1.922	1.258,	2.936					
East	0.816	0.002	2.262	1.353,	3.782					
**Constant**	**-0.404**	**0.388**	**0.668**			**0.335**	**0.172**	**1.398**		

Variables entered in the model: age, residence, region, marital status, education, social security, occupation, housing tenure, source of drinking water, the number of the household per sleeping room, smoking, alcohol consumption.

Social security status was the only socio-economic status indicator in the final models. Those who had SSK and no social security were at greater risk than those who had insurance of Emekli Sandigi (Table 3).

The source of drinking water was a significant factor in the final models. The people who used piped water, spring/public fountain and other (river, rain water etc.) were at greater risk than those who used bottled water/demijohn/PET bottled water as drinking water (Table 3).

Housing was a significant factor only in the men’s model. Men lodging/or paying no money for their housing were at more risk than those who lived in a house owned by a household member (Table 5).

### Lifestyle factors and prevalence of H.pylori infection

Smoking and alcohol consumption were associated with H.pylori infection. Regular smokers were at higher risk than non smokers. But this association did not hold for females (Tables 6 and 5). In contrast, regular alcohol consumption was a protective factor for H.pylori infection. All of those who never drink alcohol, those who had only tried in the past and the occasional drinkers had a higher risk than regular alcohol consumers (Tables 6 and 5).

**Table 6:** Lifestyle factors associated with Helicobacter pylori infection

**Lifestyle factors**	**Hp positive**		**Hp negative**			
	**n**	(%)*	**n**	(%)*	**Total**	**P**
**Smoking cigarettes** (n = 4605)						
Never	1662	(80.8)	370	(19.2)	2032	0.00
Tried at past, currently non-smoker	745	(81.6)	159	(18.4)	904	
Tried at past, currently occasional smoker	245	(82.4)	47	(17.6)	292	
Regular smoker	1190	(85.9)	187	(14.1)	1377	
**Drinking alcohol** (n = 4593)						
Regular consumer	69	(74.7)	18	(25.3)	87	0.03
Others	3762	(82.8)	744	(17.2)	4506	
**Total**	3852	(82.5)	770	(17.5)	4622	

* Weighted.

### Analysis of factors and H.pylori infection by sex

Since sex was a significant factor for H.pylori infection it was necessary to analyze factors separately for each sex. In men, age, marital status, social security status, housing tenure, type of drinking water, smoking, alcohol use and geographic region were factors. However, for women, age, marital status, social security status, type of drinking water and education were factors (Table 5).

## Discussion

So far as we know this study is the most representative one that is based on a sample derived from the population of one country, estimating the factors associated with the prevalence of Helicobacter pylori infection and using the ^13^C-UBT. Furthermore, the response rates were very high. In this study, it was produced highly significant estimates [Design effect (DEFT) = 2.01 and standard error = 0.008].

Awareness of Helicobacter pylori is little more than a decade old. Yet there have been many studies all over the world about its epidemiology. Most prevalence data have used random sampling of blood donors, clinic attendees or industrial employees; none of these groups provides a truly normal population as emphasised by Pounder [12].

Studies that have used labelled breath tests in a normal population to detect Helicobacter pylori infection are very rare. However, they are highly sensitive, specific and are also recommended by the Maastricht 2–2000 Consensus Report and by the Canadian Helicobacter Study Group Consensus conference, 2004 [7,13,14].

When comparing the rates from previous studies directly with our study, it should be kept in mind that other studies also differ from ours in terms of variation by age, type of population, type of diagnostic test and study time at which the study was done.

In TURHEP, the weighted overall prevalence of Helicobacter pylori infection was 82.5% (95% CI 81.0-84.2) with ^13^C-UBT. Helicobacter pylori prevalence has been reported to reach 70% or more in developing countries and to be less than 40% in developed countries [15-37].

There was an inverse association between age and infection in our study. Earlier studies have shown differing trends regarding age and Helicobacter pylori prevalence. Whereas Helicobacter pylori prevalence increased with age at earlier ages, there was a slight decrease in populations over 60 years of age in France and over 50 years in the other countries (Vietnam, Algeria and Ivory Coast) [15]. Infection increased up to the 40–49 age group, then decreased in analyses for Southern Brazil and Northern India [17,30]. Also, the prevalence peaked at ages 45 to 64 and dropped after the age 65 in Chile and the Czech Republic [31,37]. In Ankara (Turkey), seroprevalence was 58.4% for ages 15–19, 62.6% for ages 20–29, 67.6% for ages 30–39, 81.3% for ages 40–49 and 66.3% for over 50 years [38]. In India also the prevalence was increasing to 100% by 60 then decreasing to 80% by 70 years (n = 238, ages 3–70) and in Athens, whereas the seroprevalence was increasing from 14.2% for ages 15–24 to 67.4% for ages 55–64, it decreased to 57.9% for ages >65 [18,32]. Only in Beninese populations, in 2005 (n = 446, over 2 years old) no association was found between seroprevalence and age [28]. In contrast, some studies claimed that Helicobacter pylori prevalence increased with age [15,16,19,21,24-26,29,33-35,39-43].

We found that men in Turkey were at greater risk than women for Helicobacter pylori infection. Likewise, in Northern California, men had a higher prevalence of antibodies across all strata of race/ethnicity, age, education and income (OR = 2.0, 95% CI 1.2-3.1) [42]. Also, in Northern Ireland, infection was more common in males (60.9%) than females (55.2%, p < 0.01, Or for males versus females was 1.19 (95% CI 1.02-1.40) [35]. In Leeds (UK), Spain and Chile, it was higher in men [31,36,44]. Conversely, in some studies, which mostly had small samples, there was no difference found in Helicobacter pylori prevalence between the sexes [15-18,21,22,24-26,28,29,34,37-39],[43]. To our knowledge, only one study from Israel found that the relative risk of Helicobacter pylori infection was increased in women smokers [19]. We agree with Moayyedi-et-al. that the positive association of Helicobacter pylori with the male sex should probably not be interpreted as a direct causal relationship [36]. The reason for the possible gender difference is unclear but may relate to young boys having poorer hygiene than young girls. Because of social gender roles in Turkey, men seek less healthy facilities for toilet needs than women, and men are outdoors more than women, which brings more risks of infection. Further, men tend to participate in more of the risky behaviours such as smoking, alcohol drinking than women.

The current residential region was found to be a risk factor for H.pylori infection. In Turkey, the western areas are more developed, more crowded, better educated, and have better housing conditions; families are smaller than in the East. The reason why H.pylori infections are lowest in individuals living in the South must be related to this being a major area for growing citrus fruits. These contain high levels of Vitamin C. People in the South can eat oranges, lemons, tangerines or bitter oranges frequently and continuously or drink the juices because citrus fruits are cheap and plentiful all the year round. It is known that Vitamin C is effective in the prevention of most infections. Also H.pylori can be expected not to survive in acidic gastric conditions produced by the acidic citrus fruits. Moreover, for regular smokers the highest H.pylori prevalence may result from an interaction between tobacco and Vitamin C. In contrast, the highest H.pylori infections were found in subjects living in eastern Turkey, which has the least available citrus fruits; they cannot be grown, and snow prevents their transport for several months each year; Besides, this region is the least developed. Although in TURHEP, dietary habits and daily consumptions were not included, supportive studies are available [45-48]. Additionally, garlic is frequently used in southern Turkey. One study presented garlic as a possible protective factor for gastric lesions with H.pylori infection [46].

Some studies with small sample sizes comparing the regions are available from Turkey. H.pylori infection was found to be 73.8% in the West, 48%-81% in the Central, 60%-85.4% in the Eastern parts of the country [38,39,49-51].

In most of studies it was found that H.pylori infections were inversely related to level of education [16,22,24,34,37,42,43]. Likewise in TURHEP, the lower the education of the subjects, especially for females, the higher the risk for H.pylori infection. However, two other studies found no association [19,28].

The status of social security was a significant factor in the TURHEP study’s final model and in the models by sex. To our knowledge, this variable has not previously been used as a socio-economic status indicator in any study related with H.pylori infection. Some previous studies have presented an inverse association between H.pylori infection and family income as a socio-economic status indicator [29,38,42,43,52-54]. In TURHEP, the lower the income of the subjects, the higher the infection, but only in a univariate analysis. Some researchers have also studied the association between infection and social class/socio-economical class. In Korea in adults, the rate of infection was high and independent of socio-economic class. However, in children, it was inversely related to the socio-economic class of the child’s family [21]. In Northern Ireland, the adjusted OR of infection in subjects from manual workers relative to those from non-manual occupations was 1.7 (95% CI: 1.47-1.98) [35]. In Northern England, infection was more common in the lower social class groups [36]. In Libya, 91% of a low socio-economic class was H.pylori-positive, while those of middle and high socio-economic classes showed 53% and 57% positivity respectively [24]. In Northern India infection was not associated with socio-economic status [17].

Housing tenure, as another socio-economic indicator was found significant only in a model for males in TURHEP. In contrast, another study, showed no association between prevalence of H.pylori and type of housing (owned/rented)[21].

In TURHEP, a water- H.pylori infection association was found in the final models. This association is a question about H.pylori infection being one of the water-borne contagious diseases. This association was mentioned in many studies from different parts of the world and it has been found that there is mostly a positive significant relation [1,21,25,52]. On the other hand, no association was found in studies from Benin and Turkey [28,39].

Smoking was a significant factor for H.pylori infection in TURHEP except for the female model. Similar results have been presented in some studies [19,35-37]. However smoking was not associated with H.pylori infection in some other studies [15,16,21,23,30,34,43].

In TURHEP, regular alcohol consumption was found to be a protective factor except for the females model. Similar results have also been presented in some earlier studies [53-56]. In a EUROGAST Study, a univariate analysis showed that alcohol consumption was associated with a reduced prevalence of H.pylori, but this effect disappeared completely after adjustment in the multivariate analysis [34]. No association was found between H.pylori and alcohol use in other studies [15,21,30,35,36].

## Conclusions

In Turkey, H.pylori prevalence was found to be very high. Individuals, who were women, elderly adults, single, at high educational levels, living in southern Turkey, having social security of Emekli Sandigi, drinking bottled water, non-smokers and regular alcohol consumers, were under less risk of H.pylori infection than others.

In the TURHEP study, whereas prevalence was estimated as 82.5% (95%CI 81.0-84.2) in the adult population, age, sex, education and marital status were suggested as playing critical roles as co-factors for H.pylori infection. Social security, housing tenure and also water have dependant role. Whereas smoking, a common habit especially in men was positively associated, alcohol use, not as common as smoking, was a protective factor for H.pylori. Living in the southern region of Turkey, a citrus fruit growing area, is seen as a protective factor for H.pylori infection and was the most interesting result in TURHEP.

We have presented high quality data from normal, healthy individuals, representative of the whole country, from Turkey. The results of the TURHEP study, offer important public health implications for the prevention of H.pylori. In the future, cohort studies should be implemented to help define more significant risk factors.

## Endnotes

^a^Emekli Sandigi: The pension fund for civil servants, SSK ‘Social Security Institution’ the insurance of employee, BAG-KUR: the insurance of tradesman, artists and other freelance workers, Green-Card: limited insurance of people do not have any other insurance.

## Competing interests

The authors declare that they have no competing interests.

## Authors’ contributions

NO conceived of the study, developed the questionnaire, monitoring the survey, performed the statistical analysis and finalized the manuscript. ST participated in the study design and helped to draft the manuscript. SC participated in the study design, developed the semi-structural interview guide, and helped to draft the manuscript. All authors read and approved the final manuscript.

## Pre-publication history

The pre-publication history for this paper can be accessed here:

http://www.biomedcentral.com/1471-2458/13/1215/prepub

## Supplementary Material

Additional file 1: Figure S1 Distribution of the cities selected for sample for the Helicobacter pylori prevalence study. Click here for file

Additional file 2: Figure S2 Helicobacter pylori prevalence in Turkey by region. Click here for file
