# Supplementary material for: Prevalence and risk factors of helicobacter pylori in Turkey: a nationally-representative, cross-sectional, screening with the 13C-Urea breath test
Source: BMC Public Health. 2013 Dec 21;13:1215. doi: 10.1186/1471-2458-13-1215 (PMC3880349; doi:10.1186/1471-2458-13-1215)
Supplement: Additional file 1: Figure S1 — Distribution of the cities selected for sample for the Helicobacter pylori prevalence study. [file 1471-2458-13-1215-S1.ppt]

## Slide 1
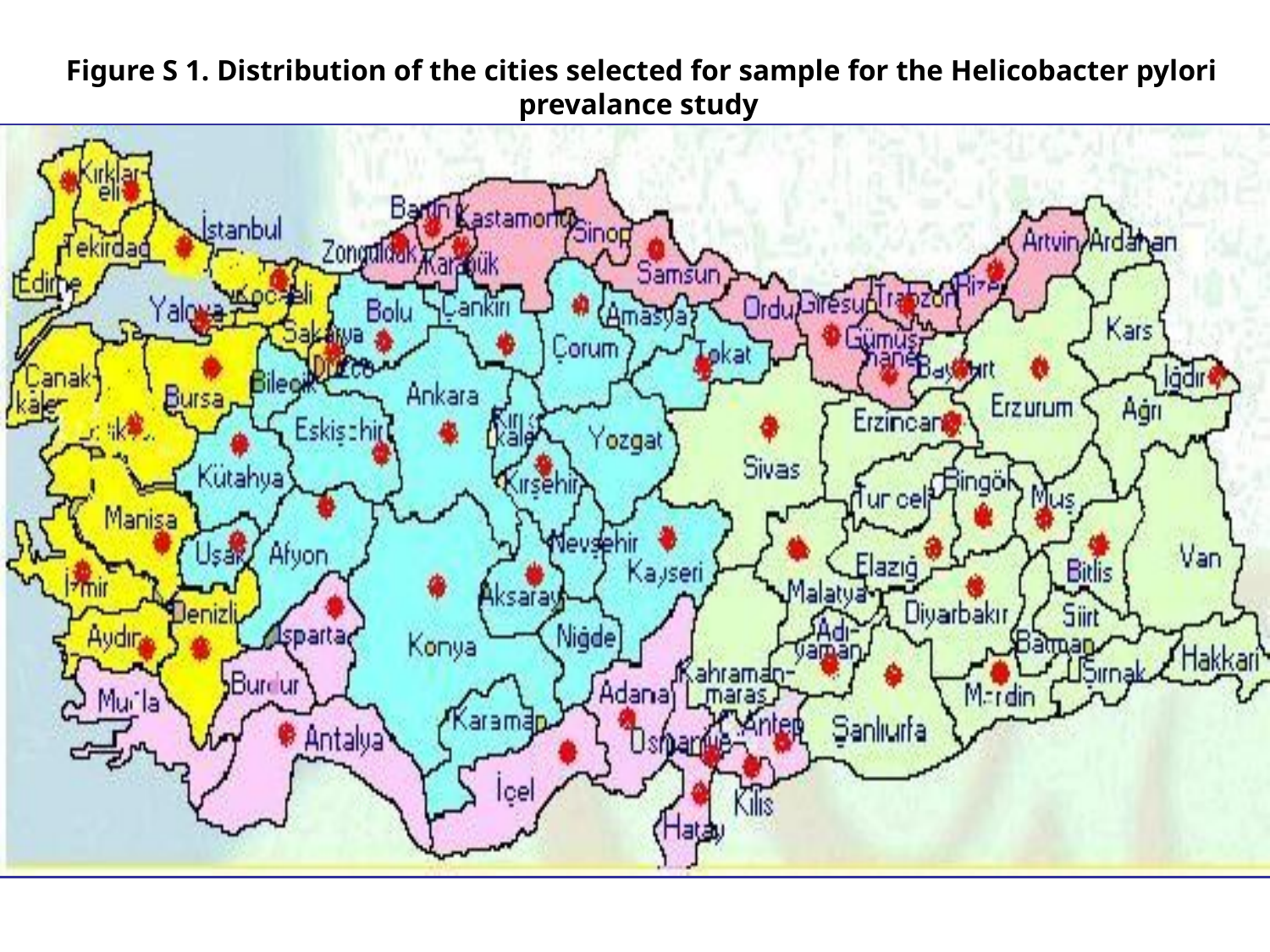

Figure S 1. Distribution of the cities selected for sample for the Helicobacter pylori prevalance study
