# Supplementary material for: Prevalence and risk factors of helicobacter pylori in Turkey: a nationally-representative, cross-sectional, screening with the 13C-Urea breath test
Source: BMC Public Health. 2013 Dec 21;13:1215. doi: 10.1186/1471-2458-13-1215 (PMC3880349; doi:10.1186/1471-2458-13-1215)
Supplement: Additional file 2: Figure S2 — Helicobacter pylori prevalence in Turkey by region. [file 1471-2458-13-1215-S2.ppt]

## Slide 1
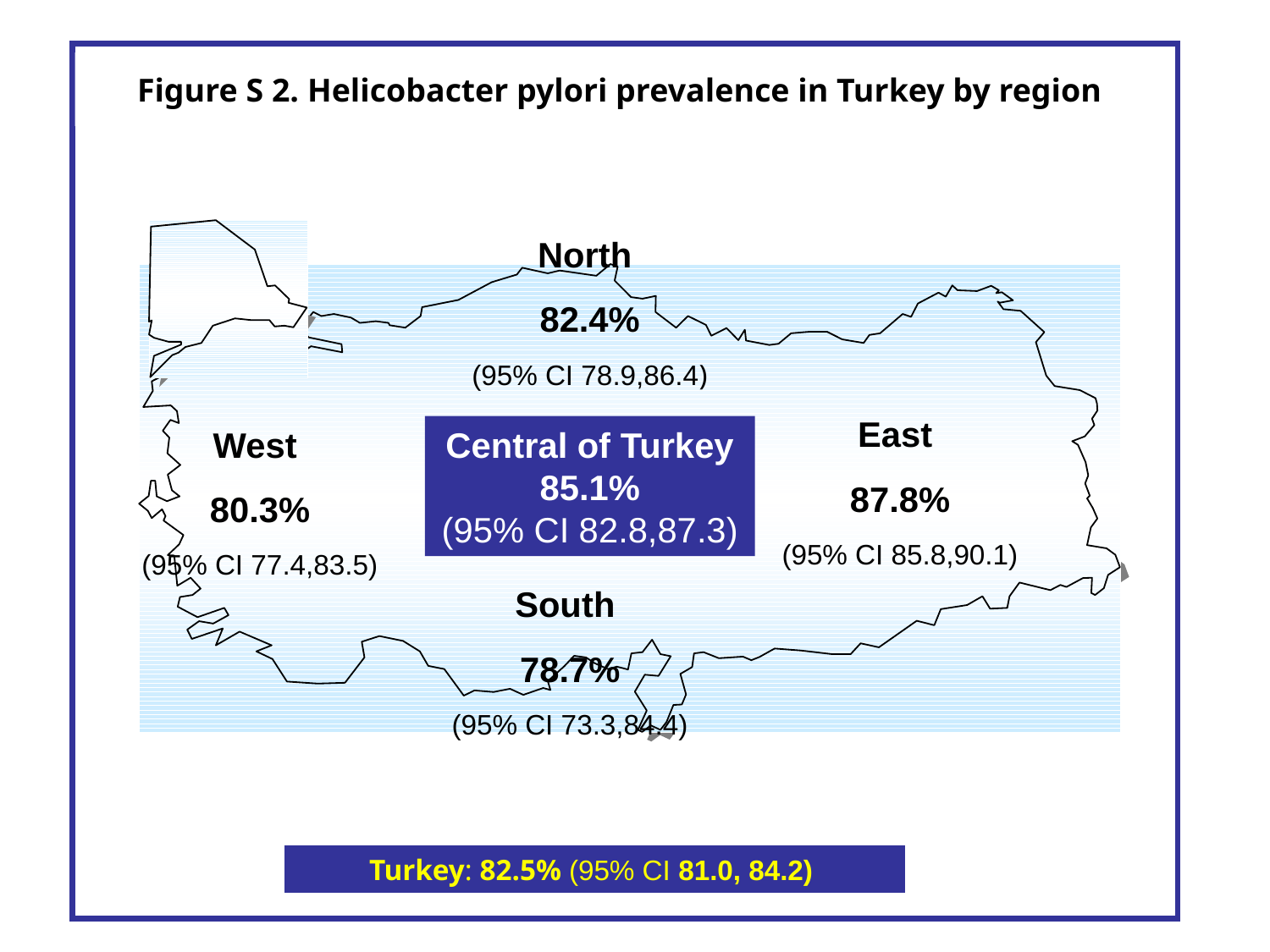

# Figure S 2. Helicobacter pylori prevalence in Turkey by region
North
82.4%
(95% CI 78.9,86.4)
East
87.8%
(95% CI 85.8,90.1)
West
80.3%
(95% CI 77.4,83.5)
Central of Turkey
85.1%
(95% CI 82.8,87.3)
South
78.7%
(95% CI 73.3,84.4)
Turkey: 82.5% (95% CI 81.0, 84.2)
